# Blood Group O Protect End-Stage Renal Disease Patients With Dialysis From Coronary Artery Disease

**DOI:** 10.3389/fcvm.2021.821540

**Published:** 2022-01-28

**Authors:** Zixiang Ye, Yaxin Wu, Yimin Tu, Mulei Chen, Yanxiang Gao, Linying Shi, Peizhao Li, Enmin Xie, Ziyu Guo, Qing Li, Xiaozhai Yu, Yike Li, Wenquan Niu, Jingyi Ren, Jingang Zheng

**Affiliations:** ^1^Department of Cardiology, Peking University China-Japan Friendship School of Clinical Medicine, Beijing, China; ^2^Graduate School of Peking Union Medical College, Chinese Academy of Medical Sciences and Peking Union Medical College, Beijing, China; ^3^Department of Cardiology, Chaoyang Hospital, Capital Medical University, Beijing, China; ^4^Department of Cardiology, China-Japan Friendship Hospital, Beijing, China

**Keywords:** coronary artery disease, end-stage renal disease, ABO blood group, Gensini score, dialysis

## Abstract

**Objective:**

Our study aims to investigate the role of the ABO blood group in the development and severity of coronary artery disease (CAD) in end-stage renal disease (ESRD) patients with dialysis.

**Methods:**

A total of 408 ESRD patients with dialysis between January 2010 and December 2020 were enrolled including 204 patients diagnosed with CAD undergoing coronary angiography for the first time, and baseline characteristics as well as Gensini score (GS) were collected. Logistic regression analysis and linear regression analysis were performed to evaluate the relation of ABO blood types to the risk and severity of CAD, respectively.

**Results:**

Blood group O frequency was significantly low in dialysis ESRD patients with CAD (25 vs. 38.24%) compared with the non-CAD patients and multivariable logistic regression showed blood group O was negatively associated with the risk of CAD [adjusted odds ratio (*OR*) = 0.33, 95% *CI* = 0.19–0.60, *p* < 0.001] as well as the GS tertiles (adjusted *OR* = 0.23, 95% *CI* = 0.11–0.49, *p* < 0.001) compared with A blood group. Blood group A, B, and AB were positively associated with the high Gensini tertile compared with O blood group (adjusted *OR* = 4.26, 95% *CI* = 2.03–8.93, *p* < 0.001; adjusted *OR* = 2.39, 95% *CI* = 1.11–5.13, *p* < 0.05; adjusted *OR* = 4.33, 95% *CI* = 1.40–13.35, *P* < 0.05). Similarly, multivariable linear regression results revealed O blood type was negatively associated with the GS (β = −26.129, 95% *CI* = −40.094 to −12.164, *p* < 0.001).

**Conclusion:**

This case-control study demonstrated that blood group O was a potential independent protective factor for the risk and severity of CAD in ESRD patients with dialysis.

## Introduction

Cardiovascular disease is a kind of serious and common disease with high morbidity and mortality (1). The prevalence of the cardiovascular disease has been continuously increasing and placing a great burden on the healthcare system of nation (2). Chronic kidney disease (CKD) is a high-incidence disease, and more than 14% of people suffered from CKD in the United States (3). Cardiovascular disease serves as the major cause of death in patients with CKD worldwide (4), because these patients are exposed to many cardiovascular disease risk factors, such as diabetes, inflammation, and oxidative stress (5). End-stage renal disease (ESRD) is the final stage of CKD with irreversible kidney function dysfunctions. It is reported that cardiovascular disease was responsible for more than half of all deaths among the patients with ESRD (6). Although there are many potential factors leading to cardiovascular disease in patients with ESRD, it has not been studied whether ABO blood groups served as an independent predictor for cardiovascular disease in ESRD groups with dialysis.

Research about the association between ABO blood groups and cardiovascular disease has been carried out for many years. Some studies reported that O blood groups had the lowest probability of thrombosis compared with non-O blood groups (7), and non-O blood groups were more likely to suffer from ischemic cardiomyopathy (8). Moreover, some reports indicated that B or AB blood groups had a higher incidence of cardiovascular disease (9), while other reports showed that A blood group was related to the incidence of coronary heart disease (CHD) and acute myocardial infarction (10, 11). However, some reports pointed out no connection between ABO blood groups and cardiovascular disease (12, 13). In all, previous studies have lacked large-scale cohort study about ABO blood groups and cardiovascular disease in patients with ESRD. Therefore, the association between blood type and CHD in patients with ESRD, as well as the severity of CHD, were studied retrospectively in our study.

In summary, the relationship between the risk and severity of cardiovascular disease and ABO blood groups in ESRD patients with dialysis is not well-researched yet. This retrospective study aimed to evaluate the association with ABO blood type and both risk and severity of coronary artery disease (CAD) in the dialysis ESRD group.

## Methods

### Study Design

This is a retrospective study to explore the relationship between ABO blood groups and the prevalence of CAD as well as the severity in patients with ESRD complying with the Declaration of Helsinki. During January 2010 to December 2020, 352 dialysis ESRD patients with CAD were consecutively acquired from China-Japan Friendship Hospital and Beijing Chaoyang Hospital, and 400 dialysis ESRD patients without CAD were randomly extracted at the same time. All patients included were with ABO blood groups information available and had undergone hemodialysis or peritoneal dialysis for at least 3 months prior to the study from January 2010 to December 2020. Patients were excluded, if they were under 18 years old, had severe hematologic disorders or liver dysfunction, pregnant, lactation, serious infectious disease, malignant disease, and incapable to consent. Finally, a total of 204 dialysis ESRD individuals with CAD were selected as the case and 204 dialysis ESRD patients without CAD were matched according to age and gender as controls. The approval was performed by the hospital ethics review committee of China-Japan Friendship Hospital and Beijing Chaoyang Hospital. All data were retrieved from the hospital information system; thus, informed consent was not acquired.

The demographic baseline characteristics and laboratory test details were extracted from all patients during hospitalization by using International Classification of Diseases, 9th Revision, Clinical Modification (ICD-9-CM) codes and hospital information system procedure codes. Agglutination techniques were used to determine the ABO blood type according to standard procedures. Hypertension was defined as the repeated blood pressure (BP) measurement more than 140/90 mmHg or on the medications for hypertension currently. Diabetes mellitus (DM) was defined as fasting glucose level ≥7.0 mmol/L (126 mg/dl) or 2-h post load glucose level ≥11.0 mmol/L (200 mg/dl), or on the medications for diabetes currently. Hypercholesterolemia was defined as low-density lipoprotein (LDL) cholesterol level ≥130 mg/dl or total cholesterol (TC) level ≥200 mg/dl, or on the medications for hypercholesterolemia currently. Echocardiograph was utilized to evaluate the left ventricular ejection fraction (EF).

### End-Stage Renal Disease

Chronic kidney disease was defined as glomerular filtration rate (GFR) <60 ml/min/1.73 m^2^ for more than 3 months with additional kidney damage markers. The concept of dialysis patients with ESRD was almost in accord with CKD Grade 5 (GFR <15 mL/min/1.73 m^2^) (14), dialysis patients with ESRD were defined as the group which was irreversible kidney function dysfunctions undergoing hemodialysis or peritoneal dialysis at least more than 3 months (15).

### Coronary Atherosclerosis and Gensini Score (GS)

The definition of CAD was more than half of stenosis in at least one major coronary branch based on coronary angiography. Non-CAD patients were defined as less than half of stenosis in any coronary branch based on coronary angiography or were not diagnosed as CAD in medical records. Gensini score (GS), which was a widely acknowledged angiographic scoring system, was performed to quantify the severity of CAD (Supplementary Table 1). According to the guide of the GS (16), the degree of stenosis score was multiplied by the lesion site score, and the final GS was the sum of all the lesion scores. Two separate doctors calculated and revised the GS blinded to the study design.

### Statistical Analysis

The sample size was evaluated according to the estimated prevalence of O blood group in patients with ESRD of 41% and the estimated decreased risk of CAD by 0.40 times in O blood population. At least 135 dialysis ESRD patients with or without CAD were required, respectively, with 1:1 control to case ratio at 95% *CI* level and 80% power.

The baseline characteristics entry and curation were performed by Microsoft Excel 2016 and software R (version 4.0.3, Vienna, Austria). STATA (Version 16.0, TX, USA) was conducted to analyze the data. Categorical variables data were presented as frequencies and percentages (%), while continuous data were shown as mean ± SD for normally distributed values or medians (interquartile ranges, IQR) for skewed distribution. Student's *t*-test or ANOVA were performed for between-group comparisons in normally distributed continuous variables, otherwise by the Wilcoxon rank-sum test. Categorical data comparisons were conducted by using the chi-square test. Univariable and multivariable logistic regression analyses were utilized to analyze the independent association between ABO blood type and CAD. Univariate and multivariate linear regression analyses were performed to analyze the role of ABO blood groups in the severity of CAD in patients with ESRD. Two-tailed *p* < 0.05 were taken to be statistically significant.

## Results

### Characteristics of Dialysis Patients With ESRD

A total of 408 dialysis patients with ESRD from China-Japan friendship hospital and Beijing Chaoyang hospital were included in this research, including 204 CAD patients and 204 non-CAD patients matched according to age and gender. Table 1 exhibits the comparisons of the baseline characteristics between dialysis ESRD patients with and without CAD. CAD risk factors, such as BMI, hypertension, hypercholesterolemia, DM, and smoking were not significantly different. Dialysis ESRD patients with CAD had significantly higher glucose, hemoglobin, platelet, and dialysis duration periods compared with non-CAD patients.

**Table 1:** Baseline characteristics of dialysis end-stage renal disease (ESRD) patients with or without coronary artery disease (CAD).

	**Total (*N* = 408)**	**CAD (*N* = 204)**	**Non-CAD (*N* = 204)**	***P*-value**
**Chracteristic**				
Age, yrs	62.4 ± 10.1	62.4 ± 10.2	62.4 ± 12.9	0.899
Gender, *n* (%)				1
Male	288 (70.6)	144 (70.6)	144 (70.6)	
Female	120 (29.4)	60 (29.4)	60 (29.4)	
**Coronary risk factor**				
BMI, kg/m^2^	23.7 ± 3.7	23.7 ± 3.5	23.8 ± 3.9	0.925
Hypertension, *n* (%)	374 (91.7)	186 (91.2)	188 (92.2)	0.864
Hypercholesterolemia, *n* (%)	348 (85.3)	173 (84.8)	175 (85.8)	0.893
DM, *n* (%)	189 (46.3)	101 (49.5)	88 (43.1)	0.226
Smoking, *n* (%)	178 (43.6)	97 (47.5)	81 (39.7)	0.133
**Laboratory test**				
Glucose, mmol/L	5.2 (4.5, 6.9)	5.5 (4.6, 7.7)	5 (4.4, 6.2)	0.021
WBC, 10^9^/L	6.5 (5.1, 8.1)	6.6 (5.4, 8.4)	6.3 (5.0, 7.8)	0.079
Hemoglobin, 10^9^/L → g/L	101 (86, 114)	105.5 (94, 117)	96 (80, 111)	0.008
PLT, 10^9^/L	167 (126, 218)	178 (145, 232)	155 (116, 205)	0.007
Uric Acid, mmol/L	359 (283, 440)	356 (276, 433)	362 (289, 453)	0.311
Fibrinogen, mg/dL	4 (3, 5)	4 (3, 5)	4 (3, 5)	0.938
Phosphorus, mmol/L	1.56 ± 0.53	1.73 ± 0.49	1.40 ± 0.51	<0.01
Calcium, mmol/L	2.23 ± 0.23	2.22 ± 0.25	2.24 ± 0.20	0.394
**Lipid profile**				
Triglyceride, mg/dL	1.5 (1, 2.2)	1.6 (1.2, 2.5)	1.3 (1, 2)	<0.01
TC, mg/dL	3.8 (3.1, 4.6)	3.4 (2.7, 4.2)	4.2 (3.6, 4.9)	<0.01
LDLC, mg/dL	2.2 (1.7, 2.8)	2 (1.5, 2.7)	2.3 (2, 2.9)	<0.01
HDLC, mg/dL	1 (0.8, 1.2)	0.9 (0.7, 1)	1.1 (0.9, 1.4)	<0.01
**Dialysis**				
Type, *n* (%)				0.009
Peritoneal dialysis	68 (16.7)	24 (11.8)	44 (21.6)	
Hemodialysis	340 (83.3)	180 (88.2)	160 (78.4)	
Duration, months	3 (1, 6)	4 (2, 8)	2 (1, 4.2)	0.008

### ABO Blood Groups Distribution and CAD in Patients With ESRD

**Figure 1:**
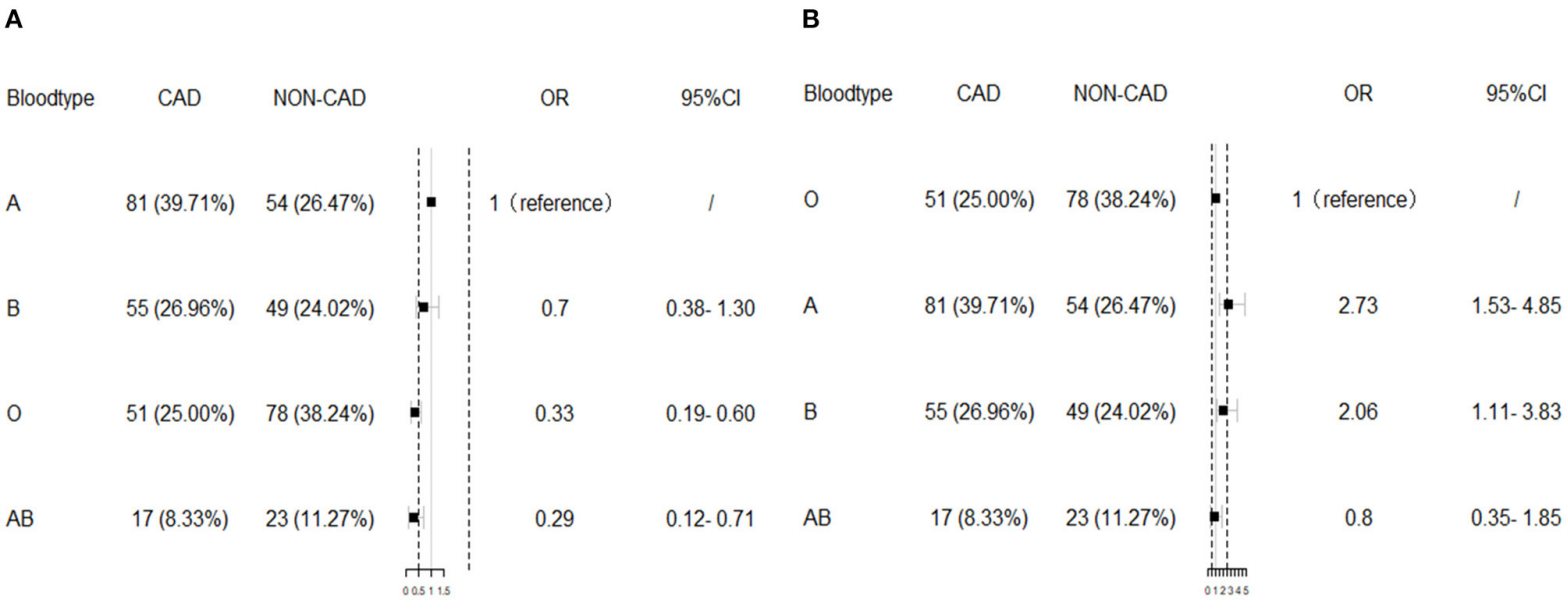
The multivariate logistic regression results. Blood group O significantly associated with low risk of coronary artery disease (CAD) compared with blood group A **(A)**, while A and B blood groups were related to high risk of CAD compared with O blood group **(B)** after adjustment of body mass index (BMI), hypertension, hypercholesterolemia, diabetes mellitus (DM), smoking, glucose, white blood cell (WBC), hemoglobin, platelet, serum phosphorus, serum calcium, dialysis type, and dialysis duration. *OR*, odds ratio.

The distribution of the ABO blood group was significantly different between ESRD patients with and without CAD (*p* < 0.01). Patients with O blood type occupied 25% in CAD group while patients with A blood group occupied 39.71%. In dialysis ESRD patients without CAD, blood group O accounted for 38.24% while blood group A accounted for 26.47%. Blood group O and AB significantly associated with low risk of CAD compared with blood group A [adjusted odds ratio (*OR*) = 0.33, 95% *CI* = 0.19–0.60, *p* < 0.001; adjusted *OR* = 0.29, 95% *CI* = 0.12–0.71, *p* < 0.01, respectively], as shown in Table 2 according to the multivariate logistic regression results after adjustment of age, gender, BMI, hypertension, hypercholesterolemia, DM, smoking, glucose, white blood cell (WBC), hemoglobin, platelet, serum phosphorus, serum calcium, dialysis type, and dialysis duration. A and B blood groups were related to high risk of CAD compared with O blood group (adjusted *OR* = 2.73, 95% *CI* = 1.53–4.85, *p* < 0.01; adjusted *OR* = 2.06, 95% *CI* = 1.11–3.83, *p* < 0.05). When the individual blood group was estimated comparing with the others, blood group A significantly increased the risk of CAD (adjusted *OR* = 2.24, 95% *CI* = 1.36–3.69, *p* < 0.01) while blood group O significantly decrease the risk of CAD in ESRD groups (adjusted *OR* = 0.49, 95% *CI* = 0.27–0.77, *p* < 0.01). Blood group B and AB were not significantly different between ESRD patients with and without CAD. The multivariate logistic regression results are visualized in Figure 1.

**Table 2:** Comparison of ABO blood group between ESRD patients with or without CAD.

**Blood type**	**CAD**	**Non-CAD**				**Adjusted**		
			**OR**	**95%CI**	***P*-value**	**Adjusted OR**	**95%CI**	***P*-value**
A	81 (39.71%)	54 (26.47%)	1			1		
B	55 (26.96%)	49 (24.02%)	0.75	0.44–1.25	0.271	0.70	0.38–1.30	0.261
O	51 (25.00%)	78 (38.24%)	0.44	0.27–0.71	0.001	0.33	0.19–0.60	<0.001
AB	17 (8.33%)	23 (11.27%)	0.49	0.25–1.00	0.052	0.29	0.12–0.71	0.007
O	51 (25.00%)	78 (38.24%)	1			1		
A	81 (39.71%)	54 (26.47%)	2.29	1.40–3.76	0.001	2.73	1.53–4.85	0.002
B	55 (26.96%)	49 (24.02%)	1.72	1.02–2.89	0.043	2.06	1.11–3.83	0.021
AB	17 (8.33%)	23 (11.27%)	1.13	0.55–2.32	0.738	0.80	0.35–1.85	0.598
A	81 (39.71%)	54 (26.47%)	1.83	1.20–2.78	0.005	2.24	1.36–3.69	0.002
Other group	123 (60.29%)	150 (73.53%)	1			1		
B	55 (26.96%)	49 (24.02%)	1.17	0.75–1.82	0.496	1.26	0.74–2.14	0.399
Other group	149 (73.04%)	155 (75.98%)	1			1		
O	51 (25.00%)	78 (38.24%)	0.54	0.35–0.82	0.004	0.49	0.27–0.77	0.003
Other group	153 (75.00%)	126 (61.76%)	1			1		
AB	17 (8.33%)	23 (11.27%)	0.72	0.37–1.38	0.320	0.5	0.22–1.12	0.090
Other group	187 (91.67%)	181 (88.73%)	1			1		

### The Correlation Between O Blood Groups and Severity of CAD in ESRD Patients With CAD

Table 3 summarized the baseline characteristics of dialysis ESRD patients with CAD in different blood groups (*N* = 204). The majority patients in the O blood group have low GS, while patients with high GS accounted for more in A blood group. Among individuals suffering from both CAD and ESRD, the GS and GS tertiles were significantly different among various ABO blood groups (*p* < 0.001). Other baseline characteristics, such as well-known CAD risk factors, laboratory test results, lipid profiles, and dialysis information were not statistically different among different ABO blood groups.

**Table 3:** Baseline characteristics of different blood group types ESRD patients with CAD.

	**A (*N* = 81)**	**B (*N* = 55)**	**O (*N* = 51)**	**AB (*N* = 17)**	***P*-value**
Gensini score	74.99 ± 46.95	64.96 ± 39.76	44.15 ± 33.34	80.65 ± 57.45	<0.001
Gensini score, *n* (%)					0.009
Low ( ≤ 36)	18 (22.2)	12 (21.8)	25 (49.0)	4 (23.5)	
Mid (37–87)	29 (35.8)	31 (56.4)	19 (37.3)	6 (35.3)	
High (≥88)	34 (42.0)	12 (21.8)	7 (13.7)	7 (41.2)	
Chracteristic					
Age, yrs	61.47 ± 10.11	62.38 ± 10.00	64.51 ± 10.58	60.29 ± 9.99	0.313
Gender, *n* (%)					0.242
Male	51 (63.0)	40 (72.7)	40 (78.4)	13 (76.5)	
Female	30 (37.0)	15 (27.3)	11 (21.6)	4 (23.5)	
Coronary risk factor					
BMI, kg/m^2^	24.12 ± 3.60	23.69 ± 3.46	23.27 ± 3.07	22.90 ± 3.85	0.412
Hypertension, *n* (%)	79 (97.5)	52 (94.5)	44 (86.3)	11 (64.7)	0.008
Hypercholesterolemia, *n* (%)	75 (92.6)	48 (87.3)	39 (76.5)	11 (64.7)	0.007
DM, *n* (%)	39 (48.1)	28 (50.9)	28 (54.9)	6 (35.3)	0.558
Smoking, *n* (%)	37 (45.7)	24 (43.6)	26 (51.0)	10 (58.8)	0.669
EF	57 (45, 64)	55 (44, 67)	60 (48, 66)	50 (40, 63)	0.499
Laboratory test					
Glucose, mmol/L	5.39 (4.31, 8.06)	5.68 (4.76, 7.94)	5.55 (4.53, 7.26)	5.62 (4.60, 6.90)	0.734
WBC, 10^9^/L	6.37 (5.58, 7.90)	6.45 (5.24, 8.77)	6.60 (5.42, 8.00)	7.10 (5.76, 10.51)	0.599
Hemoglobin, 10^9^/L → g/L	102 (92, 114)	107 (93, 118)	105 (91, 118)	110 (101, 120)	0.321
PLT, 10^9^/L	202 (150, 245)	164 (134, 189)	169 (144, 205)	195 (128, 220)	0.029
Uric Acid, mmol/L	361 (264, 430)	356 (295, 413)	352 (273, 453)	353 (270, 407)	0.959
D-dimer, mg/dL	638 (370, 1,179)	550 (375, 1,017)	450 (280, 769)	710 (340, 879)	0.345
Fibrinogen, mg/dL	4.26 (3.21, 5.26)	4.20 (3.44, 4.84)	4.10 (3.38, 4.59)	4.36 (3.57, 4.70)	0.644
Phosphorus, mmol/L	1.69 (1.46, 1.96)	1.77 (1.35, 2.12)	1.76 (1.45, 2.13)	1.78 (1.38, 1.99)	0.690
Calcium, mmol/L	2.20 (2.03, 2.34)	2.19 (2.07, 2.37)	2.25 (2.10, 2.39)	2.18 (2.03, 2.43)	0.343
Lipid profile					
Triglyceride, mg/dL	1.79 (1.25, 2.87)	1.54 (1.15, 2.11)	1.42 (1.04, 1.94)	1.51 (1.16, 1.87)	0.065
TC, mg/dL	3.47 (2.74, 4.24)	3.34 (2.80, 4.14)	3.36 (2.62, 4.09)	3.44 (2.98, 3.85)	0.912
LDLC, mg/dL	2.20 (1.73, 2.82)	1.87 (1.54, 2.63)	1.76 (1.26, 2.22)	1.88 (1.40, 2.48)	0.071
HDLC, mg/dL	0.83 (0.72, 1.01)	0.82 (0.74, 1.02)	0.93 (0.74, 1.10)	0.98 (0.84, 1.18)	0.142
Dialysis					
Dialysis type (%)					0.786
Peritoneal dialysis	11 (13.6)	7 (12.7)	5 (9.8)	1 (5.9)	
Hemodialysis	70 (86.4)	48 (87.3)	46 (90.2)	16 (94.1)	
Duration	5.82 ± 4.57	4.87 ± 4.06	5.32 ± 3.61	4.85 ± 3.64	0.563

The relationship between ABO blood groups and the severity of CAD based on the GS tertiles (low tertile ≤ 36; mid tertile 37–87; high tertile ≥88) in ESRD patients with CAD were determined *via* univariate and multivariate logistic regression analyses. After adjustment of age, gender, BMI, hypertension, hypercholesterolemia, DM, smoking, glucose, WBC, hemoglobin, platelet, uric acid, serum phosphorus, serum calcium, dialysis type and dialysis duration, blood group A, B, and AB comparing with O blood group were positively associated with high GS tertile (adjusted *OR* = 4.26, 95% *CI* = 2.03–8.93, *p* < 0.001; adjusted *OR* = 2.39, 95% *CI* = 1.11–5.13, *p* < 0.05; adjusted *OR* = 4.33, 95% *CI* = 1.40–13.35, *p* < 0.05, respectively). Blood group O compared with blood group A was negatively associated with the high GStertile (adjusted *OR* = 0.23, 95% *CI* = 0.11–0.49, *P* < 0.001). Table 4 exhibits the results of logistic regression analysis.

**Table 4:** Comparison the Gensini score (GS) of different ABO blood groups in ESRD patients with CAD.

**Blood type**				**Adjusted**		
	**OR**	**95%CI**	***P*-value**	**Adjusted OR**	**95%CI**	***P*-value**
A	1			1		
B	0.59	0.31–1.13	0.112	0.56	0.28–1.11	0.098
O	0.24	0.12–0.48	<0.001	0.23	0.11–0.49	<0.001
AB	0.95	0.36–2.52	0.915	1.02	0.34–3.03	0.977
O	1			1		
A	4.17	2.10–8.25	<0.001	4.26	2.03–8.93	<0.001
B	2.48	1.20–5.11	0.014	2.39	1.11–5.13	0.025
AB	3.95	1.39–11.20	0.010	4.33	1.40–13.35	0.011

Linear regression analysis was utilized to evaluate the relationship between O blood groups and the severity of CAD in patients with ESRD. Univariate linear regression analysis demonstrated that GS was associated with the O blood type in patients with ESRD as well as smoking (*p* = 0.025), EF (*p* = 0.042), WBC (*P* = 0.036), and fibrinogen (*p* = 0.004) (Supplementary Table 2). In the multivariate linear regression analysis, O blood group compared with non-O blood groups was associated with GS after the adjustment of smoking, fibrinogen, DM, gender, and WBC (β = −26.866, 95% *CI* = −40.227 to −13.504, *p* < 0.01, Model 5, Supplementary Table 3). In the final multivariate linear regression model (Model 6, Supplementary Table 3), after adjusting for smoking, fibrinogen, EF, hypercholesterolemia, age, gender, BMI, hypertension, DM, hemoglobin, WBC, PLT, uric acid, serum phosphorus, serum calcium, dialysis type, dialysis duration, and O blood type was negatively associated with increased GS (β = −26.129, 95% *CI* = −40.094 to −12.164, *p* < 0.001).

## Discussion

This was the first study to demonstrate that there was an association between ABO blood type and cardiovascular disease in ESRD patients with dialysis in the Chinese group and the relationship among various ABO blood groups with the severity of CAD estimated with Gensini score. The O blood group was a potential independent protective factor for the risk and severity of CAD in dialysis patients with ESRD compared with blood group A. Blood group A, B, and AB were potential risk factors compared with blood group O for the severity of CAD in dialysis patients with ESRD.

Our conclusions are the same as the results of most previous studies conducted in the general population without kidney disease and cast doubt on the results of other studies. The present study illustrated that blood type O was negatively related to CAD risk compared with blood type A. Similarly, several studies suggested that non-O blood groups confer a higher risk of cardiovascular events. A prospective study in 1990 illustrated A blood group was associated with the risk of ischemic heart disease in British men (11). The same relationship was detected in the Italian population (8) and Taiwanese young men (17). Recently, blood type O was reported to have a lower risk of CAD incidence compared with non-O groups in a meta-analysis that included two prospective cohort studies (18). However, a cross-sectional study enrolled 2,026 individuals excluded any significant association between the distribution of ABO blood groups in CAD groups undergoing CABG in the Iranian general population. They also pointed out that no significant difference in frequency of cardiac risk factors among different blood groups in CAD patients (19). However, the patients enrolled in that study had been undergoing coronary artery bypass graft (CABG) which may cause selection bias to the result to some extent. The correlation between ABO blood type and the severity of CAD remains ambiguous especially in patients with ESRD. A cohort study that enrolled 2,919 Chinese patients with CAD suggested that there was a correlation between the ABO blood group and the severity of CAD (20) whose conclusions were the same as another large cross-sectional study (21). However, an observational study reported that in 646 Croatian patients with CAD, the association between the ABO and the extent of coronary atherosclerosis evaluated by the number of affected coronary arteries and GS cannot be observed (12). Our study suggested that the O blood group significantly associated with the decreased severity of CAD delivered from ESRD patients with dialysis in China providing clinical evidence to verify the relationship between blood type and severity of CHD further.

Several risk factors have proven the predictive value for the occurrence of cardiovascular disease in patients with ESRD. Anemia was a common complication in patients with ESRD. It is reported that the decline of hemoglobin level caused left ventricular hypertrophy through both direct and indirect mechanisms leading to the cardiovascular death in patients with ESRD (22). Another prospective cohort study between 1982 and 1991 showed that each 1 g/dl decline in hemoglobin level was independently related to the occurrence of left ventricular dilatation and the echocardiographic cardiac disease in patients on dialysis (23). In ESRD patients on peritoneal dialysis, thrombocytosis also appeared to be relevant to the severity of CAD and peripheral arterial disease assessed by Severity Index (24). A retrospective study in the Chinese non-dialysis patients with CKD concluded that serum uric acid level as a category variable was an independent risk factor for severe arterial stenosis and was associated with the risk of CAD as a continuous variable (25). Disturbance of the mineral metabolism particularly for biomarkers, such as serum calcium and phosphorus, can multiple cardiovascular disease and vascular calcification. In pediatric patients with CKD, a high-level calcium-phosphorus product predicted high carotid artery intima-media thickness and poor diastolic function (26). A 13-year pre-ESRD care registry study showed that low calcium and high phosphorus (Ca-P) trajectory positively associated with the acute coronary syndrome as well as accelerated progression in patients with CKD (27). A comparative study including 21 patients with continuous ambulatory peritoneal dialysis (CAPD) illustrated that fibrinogen levels, as a cardiovascular risk factor, were positively correlated with CAD risk in the early stage of patients with CAPD (28). Hypoalbuminemia was proved to be an adverse prognostic factor in the dialysis population for cardiac disease due to serving as a reflection of malnutrition according to a cohort of 432 patients with ESRD (29). Further, high-sensitivity C-reactive protein (CRP) served as a reflection of residual inflammatory risk which has been reported to be associated with both CKD and cardiovascular disease (30). In our study, the multivariate logistic and linear regression adjusted a variety of risk predictors, such as age, gender, BMI, hypertension, hypercholesterolemia, diabetes, smoking, glucose, WBC, hemoglobin, platelet, uric acid, serum phosphorus, serum calcium, dialysis type, and dialysis duration. Due to the limitations of retrospective research, several possible predictors could not be included in the study, such as albumin. However, these unincluded factors would not have a significant reversible impact on the results.

Many studies had reported that more severe coronary atherosclerosis was associated with an increased risk of poor outcomes (31). The GS system increases with coronary atherosclerosis complexity reflecting the risk of cardiovascular events based on coronary angiography (32). Compared with the SYNTAX score, this score is relatively easier to calculate and has a great prognosis value at the same time (33). In this study, we combined the GS system with cardiovascular risk factors to investigate the relationship between ABO blood type and severity of CAD in the ESRD patients with the best prediction information of cardiovascular prognosis, suggesting that the O blood group tends to be at low risk.

The definitive pathogenic mechanism by which ABO blood groups affect the cardiovascular disease is still unclear, but some potential mechanisms have been proposed. One of the plausible assumptions was the pro-thrombotic state related to ABO blood groups. The positions of ABO blood group genes are close to the von Willebrand factor (VWF) gene sites, which is a crucial hemostatic factor. Studies have pointed out that ABO antigen can affect the expression of VWF (34) leading to non-O blood groups having higher plasma levels of VWF compared with the O blood group (35). VWF in the plasma has the ability to regulate the function of platelets including tethering and adhesion together with ADAMTS13 (36, 37). The imbalance levels of ADAMTS13 and VWF in circulation will lead to the occurrence of cardiovascular disease (38). The ADAMTS13 locus is located near the ABO locus which might explain this phenome. However, one previous meta-analysis illustrated no significant association between the ABO serotype marker allele and ADAMTS13 (39). Another hemostatic factor whose genomic regions are strongly related to ABO is coagulation factor VIII (FVIII) (40). Factor VIII circulates bound to VWF generating a fibrin blood clot (41). A previous study demonstrated that among African Americans, FVIII is strongly associated with the risk of CHD and total mortality with the influence of gene ABO and VWF (42). However, after adjusting ABO genetic variants, no significant correlation between ABO CpG sites and FVIII existed. Another cross-sectional observational study pointed out that ABO blood type exerted positive interrelated effects on both VWF and FVIII proteins occurred in non-O blood groups (43). ABO blood groups relate with some lipid profiles, such as TC and LDL-C (44) and inflammatory biomarkers genetically (45). One genetic study illustrated ABO locus have ability to independently influence soluble E-selectin level to intermediate TG/HDL-C ratio which provides evidence for the impact of ABO blood type on atherosclerotic cardiovascular disease (46). At the same time, ABO antigen (A, B, and H determinants) is not only expressed on red blood cells, but also widely expressed on endothelial cells, epithelial cells, T cells, and B cells (47). Participation in leukocyte adhesion may be related to inflammation in the process of cardiovascular disease (48). Besides, ABO antigens may play an important role in cell–cell interactions which are regarded as traditional cardiovascular risk factors (20, 49).

There were some limitations in this study. First, the influence of race should be considered. Our research is limited to local hospitals in Beijing bringing about inevitable selection bias. Although patients from all over the country were accepted, they could only represent the Chinese population instead of the world population. Second, some CHD patients without clinical manifestations may be missed diagnoses and not have undergone coronary angiography. Therefore, they were likely to be mistakenly included in the control group, which will cause bias in the results. However, based on the clinical experience, patients with both CHD and advanced kidney disease generally have obvious clinical symptoms, and the base of missed diagnoses was small, which has little impact on the results. Next, the GS was calculated by two different doctors with systematic errors existence. However, the error has little effect on the results due to the doctors who have completed the score were all trained in a unified system and were blinded to the study design. In addition, the specific pathogenic mechanism was unable to explain fully based on our clinical research, but this study renewed the knowledge about the prediction of the presence and severity of CAD in dialysis patients with ESRD. Furthermore, prognosis and survival status of the patient will be followed-up to provide greater clinical value in the future, since the data in this study were extracted from the hospital information system and clinical outcomes were unavailable.

Albeit the limitations mentioned above, the strengths of this study were the novelty to exhibit that blood group O serves as an independent protect factor for the risk rate of CAD as well as severity in dialysis patients with ESRD in China, a group with poor prognosis as long as CHD was combined.

Overall, a significant association between ABO blood groups and CAD in dialysis patients with ESRD was observed. Besides, blood group O significantly decreased the risk of CAD and the severity of CAD in dialysis ESRD groups compared with blood group A. Non-O blood groups were positively associated with the severity of CAD in dialysis patients with ESRD. Our study demonstrated that blood group O may play a potential protective role in the development and severity of CAD in ESRD patients with dialysis. Further prospective and large-scale studies are warranted on the national territory to evaluate the role of blood type in identifying ESRD patients with dialysis at the risk of developing CHD or other cardiovascular complications. In addition, the underlying pathogenic mechanisms are needed to explore in the future.

## Data Availability Statement

The data that support the findings of this study are available from the corresponding author upon reasonable request.

## Author Contributions

JZ and ZY contributed to the study design. ZY, YW, YT, QL, and JR contributed to data collection, manuscript writing, data processing, and figure mapping. MC, YG, and LS contributed to data proofread. WN, XY, and PL contributed to formal analysis and writing—original draft preparation. ZY, YL, EX, and ZG contributed to review and editing. All authors have read and agreed to the published version of the manuscript.

## Funding

This work was supported by the National Natural Science Foundation of China (91639110), the National Key Clinical Specialty Construction Project (2020-QTL-009), and the Beijing Natural Science Foundation (7172195).

## Conflict of Interest

The authors declare that the research was conducted in the absence of any commercial or financial relationships that could be construed as a potential conflict of interest. The handling editor YZ declared a shared affiliation with one of the authors ZY at time of review.

## Publisher's Note

All claims expressed in this article are solely those of the authors and do not necessarily represent those of their affiliated organizations, or those of the publisher, the editors and the reviewers. Any product that may be evaluated in this article, or claim that may be made by its manufacturer, is not guaranteed or endorsed by the publisher.
